# Fast Solution Synthesis of NiO-Gd_0.1_Ce_0.9_O_1.95_ Nanocomposite via Different Approach: Influence of Processing Parameters and Characterizations

**DOI:** 10.3390/ma14123437

**Published:** 2021-06-21

**Authors:** Jorge Durango-Petro, Christopher Salvo, Jonathan Usuba, Gonzalo Abarzua, Felipe Sanhueza, Ramalinga Viswanathan Mangalaraja

**Affiliations:** 1 Advanced Ceramics and Nanotechnology Laboratory, Department of Materials Engineering, Faculty of Engineering, University of Concepcion, Concepcion 4030000, Chile; jusuba@udec.cl (J.U.); gabarzua@udec.cl (G.A.); 2 Faculty of Chemical Sciences, University of Concepcion, Concepcion 4030000, Chile; 3 Departamento de Ingeniería Mecánica, Facultad de Ingeniería, Universidad del Bío-Bío, Concepción 4030000, Chile; csalvo@ubiobio.cl; 4 Instituto de Materiales y Procesos Termomecanicos, Facultad de Ingeniería, Universidad Austral del Chile, Valdivia 5090000, Chile; felipe.sanhueza01@uach.cl; 5 Technological Development Unit (UDT), University of Concepcion, Concepcion 4030000, Chile

**Keywords:** NiO-GDC nanocomposites, combustion synthesis, microstructure, rietveld refinement, properties

## Abstract

The synthesis of the nickel oxide-gadolinium doped ceria (NiO-GDC with 65:35 wt. %) nanocomposite powders with a stoichiometry of Gd_0.1_Ce_0.9_O_1.95_ was performed via fast solution combustion technique; using three different mixing methods: (i) CM (metal cations in an aqueous solution), (ii) HM (hand mortar), and (iii) BM (ball milling). The nanocomposite powders were calcined at 700 °C for 2 h and characterized by Transmission Electron Microscopy (TEM), X-ray fluorescence (XRF), and X-ray Diffraction XRD. The TEM and XRD analyses evidenced the well-dispersed NiO and GDC crystallites with the absence of secondary phases, respectively. Later, the calcined powders (NiO-GDC nanocomposites) were compacted and sintered at 1500 °C for 2 h. The microhardness of the sintered nanocomposites varies in accordance with the synthesis approach: a higher microhardness of 6.04 GPa was obtained for nanocomposites synthesized through CM, while 5.94 and 5.41 GPa were obtained for ball-milling and hand-mortar approach, respectively. Furthermore, it was observed that regardless of the long time-consuming ball-milling process with respect to the hand mortar, there was no significant improvement in the electrical properties.

## 1. Introduction

Solid oxide fuel cells (SOFCs) are electrochemical devices that convert chemical energy directly into electrical energy by redox reactions. Currently, this technology is a potential alternative solution for the energy crisis and environmental pollution, creating a link to move from fossil fuels to clean energy generation [1,2,3]. Unlike battery-type electrochemical cells, fuel cells are open systems that can be continuously powered, exhibiting similarities with conventional combustion systems [4,5]. Because SOFCs operate at high temperatures (between 700 °C and 1000 °C), they require highly resistant materials, which must be able to provide an expected lifetime. Therefore, the electrodes must not react with the electrolyte material and the gaseous environment. Furthermore, the microstructure of the electrode should (i) not evolve and (ii) facilitate the gas transport to the active reaction sites [6,7]. In general, these aspects are determined by the composition and microstructure of the electrode. The latter is mainly established by the nature of the starting powders and the manufacturing technique used [8].

Firstly, to improve the market competitiveness and reliability of SOFCs, reducing the material cost and simplifying the fabrication process can enhance their production on a larger scale. Lowering the operating temperature of the cells, using the intermediate temperature (500–700 °C) SOFCs (IT-SOFCs), could be a beneficial solution for SOFCs from the viewpoint of reducing the material cost and improving their durability. A significant reduction in the temperature translates into an increase in thermodynamic efficiency, system reliability, performance, and cell durability [9,10]. In this regard, ceramic materials with fluorite structure, such as aliovalent or isovalent cations doping cerium oxide are common as an electrolyte, exhibiting higher oxide ion conductivity than Ytria Stabilized Zirconia (YSZ) in the temperature range of 500–700 °C [11]. Among rare-earth dopants, cerium oxide doped with gadolinium (GDC) and doped with samarium (SDC) are widely recorded as the best materials for IT-SOFCs, due to their high conductivity and low activation energy [12,13,14,15].

Although electrodes should possess high electrocatalytic activity, the electronic and ionic conduction pathway should also be maximized to increase the active reaction site, i.e., the triple phase boundary (TPB) where the gas (fuel/oxygen), ionic conductor, and catalyst are attached [16,17,18,19,20,21]. In this sense, adoptions of the electrodes consisting of a mixed ionic-electronic conductor (MIECs) under reducing conditions bring an advantage to enlarge the active sites for the reactions, compared with electronic conductor electrodes where TPB is restricted to the electrolyte layer surface. The most popular or conventional composite electrodes consist of a metallic catalyst and an ion conductor. Nickel (Ni) has been widely used as an anode material for many years due to its low cost, good chemical stability, and excellent electrocatalytic activity [22,23]. Nickel/yttria-stabilized zirconia (Ni-YSZ) and nickel/gadolinium doped ceria (Ni-GDC) have been commonly used because they combine the catalytic activity and the high electric conduction of nickel with the ionic conductivity of the electrolyte phase. Furthermore, the ionic conductor reduces the thermal expansion coefficient (TEC) and helps to solve the trend of transition metals to change their microstructure at high temperatures and under reducing conditions.

Both micro- and macro-structural effects are the crucial issue; therefore, the influence of initial powder morphology, reduction and sintering temperatures, volumetric composition, and mechanical processing has been widely studied [9,24,25,26,27,28,29,30,31,32]. On the other hand, the cermet-type anodes have shown volumetric changes during the reduction–oxidation cycles (NiO→Ni), which result in a concentration of stress in the cell and give rise to possible mechanical failures [33].

Many efforts to improve the electrochemical performance and stability of the microstructure of Ni-GDC cermet have been widely studied [9,25,28,29]. However, most of these studies use the conventional ball milling method to obtain the precursor NiO-GDC nanocomposite. The development of cost-effective novel synthesis methods can produce the powders with tuned electrode microstructures and the tools to assess the efficiency of processing steps are crucial to improve the electrode performance for the low-intermediate operating temperatures. Only a few works have studied the synthesis of NiO-GDC nanocomposite using one-step or one-pot methods to improve the dispersion and to get the homogenous phases [34,35,36,37,38,39]. In this way, the solution combustion synthesis, which has been used to prepare a variety of technological applications with the desired composition and structure [40,41,42,43], is a good choice because of its simplicity and cost-effectiveness, and the powder quality of the product, which makes it easy and industrially scalable for the solid oxide fuel cell applications.

Therefore, in this work, the whole precursor materials are synthesized by the fast solution combustion method. Nanocomposite powders of NiO-GDC (65:35 wt. %) were obtained using the CM method, where the cations (Ni^2+^, Gd^3+^, and Ce^4+^) from the metal nitrates were dissolved in an aqueous solution and then directly combusted in just one step within a few minutes to obtain the well-dispersed and homogeneously tuned nanostructures. On the other hand, NiO-GDC (65:35 wt. %) nanocomposite powders are fabricated from the NiO and GDC phases, by two conventional methods. The first one, with the longer mixing time (72 h) is based on the ball milling (BM) and the second one, with the less mixing time (few minutes) is based on the hand mortar (HM). To study the effect of the starting NiO-GDC (65:35 wt. %) nanocomposite powders on the fabrication of solid oxide fuel cells as anode materials, the properties of the nanocomposites synthesized through the above-mentioned methods (CM, BM, and HM) were comparatively investigated and reported.

## 2. Materials and Methods

### 2.1. Nanocomposite Powders Processing and Their Characterization

Nickel (II) nitrate hexahydrate (Ni(NO_3_)_2_·6H_2_O), cerium (III) nitrate hexahydrate (Ce(NO_3_)_3_·6H_2_O), gadolinium (III) nitrate hexahydrate (Gd(NO_3_)_3_·6H_2_O), and citric acid (C_6_H_8_O_7_) were used as the starting materials for the NiO-GDC (65:35 wt. %) nanocomposite powders preparation (all reagents were purchased from Sigma Aldrich, St. Louis, MO, USA). The typical synthesis procedure of nanocomposites through different methods are as follows:

(a) CM method (metal cations in aqueous solution mixing): the stoichiometric proportions of nickel (II) nitrate hexahydrate (Ni(NO_3_)_2_·6H_2_O), cerium (III) nitrate hexahydrate (Ce(NO_3_)_3_·6H_2_O), gadolinium (III) nitrate hexahydrate (Gd(NO_3_)_3_·6H_2_O), and citric acid (C_6_H_8_O_7_) were dissolved in distilled water to form a homogenous solution. Then, the homogeneous solution was transferred into an alumina crucible and placed inside a preheated furnace (Nabertherm, LT 40/12, Lilienthal, DE) for few minutes at 500 °C to complete the combustion reaction. After the combustion process, a foam-like fluffy material was achieved, which was gently crushed and ground using mortar and pestle to yield the corresponding NiO-GDC nanocomposite powder.

(b) HM method (conventional hand mortar mixing): the stoichiometric proportions of Ni(NO_3_)_2_·6H_2_O and C_6_H_8_O_7_ were dissolved in distilled water to form a homogenous solution. Simultaneously, as a parallel reaction, the stoichiometric proportions of Ce(NO_3_)_3_·6H_2_O, Gd(NO_3_)_3_·6H_2_O, and C_6_H_8_O_7_ were dissolved in distilled water to form a homogenous solution. Similar to CM, the solutions were transferred to two different alumina crucibles and placed inside a preheated furnace at 500 °C for few minutes to complete the combustion reaction. After the combustion process, two different foams were achieved, which were gently ground separately to obtain NiO and GDC powders. Then, the NiO-GDC nanocomposites were prepared from the mixture of NiO and GDC powders, with a weight percentage of 65:35, respectively.

(c) BM method (conventional ball milling mixing): the preparation of the nanocomposite powders by this method is similar to the HM method until the combustion reaction occurs. After the combustion process, the two different foams achieved were ball milled (WiseMix^®^ Ball Mill, 5 mm ZrO_2_, 200 rpm) for 72 h using ethanol as medium and then dried. Then, NiO-GDC nanocomposite was prepared from the mixture of NiO and GDC powders, with a weight ratio of 65:35, respectively.

To investigate their physiochemical properties, the as-synthesized NiO-GDC (65:35 wt. %) nanocomposite powders were separately subjected, through different (CM, HM, and BM) methods, to post-calcination treatment using a furnace (Nabertherm HT 16/16, Lilienthal, DE) at 700 °C for 2 h. The calcined powders were denoted as CM-p, HM-p, and BM-p, respectively, in an accordance with their synthesis methods. The structural information of the NiO-GDC calcined powders was obtained using a X-ray diffractometer (Bruker AXS, D4 Endeavor, Bremen, DE), with 40 kV, 20 mA y 0.1542 nm Cu-K_α_ and XRD data collection in the angular range 20°≤2θ≤80° at 0.02°/step and 1 s/step to quantify the phases and the elements present in the NiO-GDC calcined powders and pure phases. The specific surface areas were calculated using the BET method from the nitrogen adsorption isotherms at the temperature of liquid nitrogen on a Micromeritics apparatus (TriStar II 3020, Norcross, GA, USA), the X-ray fluorescence (XRF) analysis was carried out with the Rigaku ZSX PRIMUS II spectrometer, TX, USA, for this purpose, approximately 10 g of each powder was compacted by applying a uniaxial pressure of 300 MPa to form the disc (pellet) samples. The semi-quantitative analysis was performed with SQX software provided by Malvern Panalytical Ltd, TX, USA The microstructural information was obtained from the Rietveld refinements of the XRD patterns using the software materials analysis using diffraction (MAUD) [44,45,46]. A corundum reference sample (A13-B73 provided by Bruker, Bremen, Gemany) was used as an external standard for determining the instrumental broadening [47]. The powder particle size and selected area electron diffraction (SAED) patterns were obtained by using transmission electron microscopy (TEM) with the JEOL 2010F, Boston, MA, USA.

### 2.2. Sintering and Characterization

To study the effect of the nanocomposite powder preparation methods on the bulk properties for SOFCs anodes substrate, the NiO-GDC calcined powders were uni-axially compacted using a die with 13 mm in diameter by applying a pressure of 300 MPa, producing discs of 13 mm in diameter and ~3–4 mm in width. Then, the discs were sintered in an air atmosphere at 1500 °C for 2 h with a heating and cooling rate of 3 °C·min^−1^, which were comparable conditions to the synthesis of anode nanocomposites. The sintered discs were denoted as CM-d, HM-d, and BM-d, respectively, in accordance with their synthesis methods. The hardness values of the sintered discs were obtained using a *Struers* micro-hardness tester under a load of 9.807 N (HV1) applied for 10 s. In this test, ten indentations were made on the samples at room temperature. The Vickers hardness was determined by the ratio of the applied load via a geometrically defined indenter to the contact (projected) area of the resultant impression using Equation (1):

(1) $$\mathrm{Hv}=1854.4\;\frac{P}{d^{2}}$$

where *P* is the applied load (kg) and ‘*d*’ is the indentation diagonal length (mm). In accordance with the ASTM E373 standard, the relative densities of the sintered discs were determined with help of the Archimedes method using the double-distilled water. The microstructure of the consolidated samples was analyzed by using the scanning electron microscope (SEM, JSM-6380LV, Chicago, IL, USA) technique. The SEM images were taken by a back-scattering electron detector (BSE), and the NiO and GDC phases were distinguished by the brightness values of the phases and the image processing was conducted by open-license ImageJ (Fiji) software [48]. The XRD patterns of the samples in the range of 20–80° diffraction angle were obtained using a (Bruker AXS, D4 Endeavor, Bremen, Gemany), with 40 kV, 20 mA, and 0.1542 nm Cu-K_α_ and XRD data collection at 0.02°·s^−1^. The microstructural information was obtained from the Rietveld refinements of the X-ray diffraction patterns using the software materials analysis using diffraction (MAUD) [44,45,46] and a Corundum reference sample (A13-B73, provided by Bruker, Bremen, Germany) provided by Bruker was used as an external standard for determining the instrumental broadening [47]. The electrical properties of NiO-GDC composites were studied by the impedance spectroscopy in an air atmosphere using the sintered disc specimens of the powders prepared through the three methods. The impedance spectra were obtained from 300 to 700 °C using a potentiostate/galvanostate with the two-probe configuration (BP-300, Seyssinet-Pariset, France). The frequencies evaluated were in the range from 1 Hz to 3 MHz with a test signal amplitude of 500 mV. Before the electrical measurements, the silver paste was deposited on both faces of the discs and curing at 500 °C for 2 h.

## 3. Results

### 3.1. Characterization of the Nanocomposite Powders

It is well-known that the intensity of the energy associated with each transition of the electrons is proportional to the concentration of the elements present. Based on this principle, the mol. % of cerium and gadolinium in GDC as well as the wt. % of NiO and GDC in NiO-GDC nanocomposites results corresponding to the X-ray fluorescence (XRF) are reported in Table 1. The NiO and GDC phases are closer to their stoichiometric composition value, which is consistent with the initial stoichiometry used (65:35 wt. %, respectively). On the other hand, the stoichiometric ratio in moles between gadolinium (Gd) and cerium (Ce) in each nanocomposite (CM-p, HM-p, and BM-p) is very close to the stoichiometric value of 0.1 and 0.9 mol. %, respectively. All the XRF results were calculated by eliminating some traces of impurities present in the XRF spectrum, which was attributed as the margin of error of the semi-quantification (not exceed 0.3 wt. %) and mostly correspond to the silicon and aluminum oxides materials that were always present in the structure of the furnaces.

**Figure 1:**
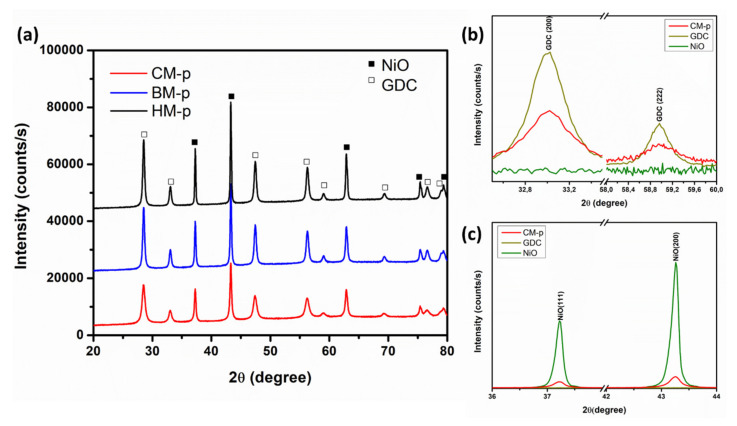
(**a**) XRD patterns of the NiO-GDC calcined powders obtained by CM, BM, and HM methods; (**b**,**c**) enlarged views of selected diffraction peaks of the CM-p for GDC and NiO phase, respectively, (p represents the calcined powder).

The XRD patterns of the NiO-GDC calcined powders obtained by the different methods are shown in Figure 1. No traces of the phases containing carbon, nitrogen, or hydrogen were observed; therefore, it is assumed that these elements were eliminated during the synthesis and calcination processes. The phases present in these diffraction patterns were identified by the search-match technique using the Joint Committee on Powder Diffraction (JCPDS ) standards database. For all the samples, the only crystalline phases presented are NiO (JCPDS card No.: 78-0643) and GDC (Gd_0.1_Ce_0.9_O_1.95_; JCPDS card No.: 75-0161). These phases belong to the Fm-3m spatial group and have simple cubic and fluorite structures, respectively. The metallic nickel (Ni) and gadolinium oxide (Gd_2_O_3_) phase peaks were not observed, which suggests that all the Ni is in nickel oxide (NiO) form and Gd^3+^ should replace Ce^4+^ cations in the cerium oxide lattice in a substitutional position to form GDC (Gd_0.1_Ce_0.9_O_1.95_).

As the CM method consists of placing all the metal cations in the same solution and combustion to guarantee an atomic level of mixing, the XRD was used to study the possibility of undesirable doping. For this purpose, an internal standard (silicon) was introduced into the samples during its preparation for the XRD tests, and subsequently, any displacement resulting from the systematic error was corrected. However, corrected diffraction patterns were analyzed and compared with that corresponding to NiO and GDC pure, and no appreciable changes were observed regarding the position of the diffraction peaks for GDC and NiO (Figure 1a,b), which is similar to the previously reported results by using the sol-gel method [34,36]. We assumed that Ni^2+^ did not enter into the GDC lattice, because the reflection peaks did not present displacement at greater angles (Ni^2+^ should replace a substitutional position of Ce^4+^), and lower angles (Ni^2+^ does not enter into the tetrahedral spaces of the fluorite structure, which would increase the GDC lattice parameters), as was previously reported for similar NiO-GDC nanocomposites calcined at 600 °C [39]. All these observations indicate the absence of the formation of a solid solution between the GDC and NiO phases. However, it is reported that at 800 °C, the (3 1 1) reflection peak of NiO appeared, due to the possible intercalation of any Ni cation inside the GDC lattice. In this work, it has been resolved by the fast solution combustion method during the calcination process at 700 °C, or the NiO existed as a separate phase because of the high temperature generated during combustion. The quantitative phase analysis is logically the next step after a qualitative examination of the crystalline phases to evaluate the influence of the different processing methods used to obtain the NiO-GDC nanocomposites.

The Rietveld method has been widely used for the determination of relatively accurate quantitative analysis and microstructural parameters (crystallite size and microstrain). To account for the microstructure of the phases, the profile fittings were performed by considering the Delft line broadening and isotropic size–strain models implemented in the MAUD software. The peak shape was assumed as a pseudo-Voigt function with asymmetry. The results of the refinements for these patterns using the room temperature crystal structure information of the phases previously identified in the qualitative analysis are shown in Table 2. The consistent pattern fittings obtained are reflected in the low figures of merit of the Rietveld refinements, in which the R_wp_ and GOF values are smaller than 10% and around 1, respectively. The phase quantification values obtained by the refinements of all samples (nanocomposites) were consistent with the initial proportions in which the NiO-GDC calcined powders were prepared (NiO-GDC 65:35 wt. %). The calculated lattice parameters were 4.1781 and 4.1780 Å for NiO and 5.4169 and 5.4203 Å for GDC in the NiO-GDC nanocomposite obtained by CM and BM, respectively. By comparing with the respective lattice parameters of the JCPDS pattern previously mentioned (NiO = 4.1790 Å, GDC = 5.4180 Å), it is confirmed that no solid solution formation was presented between the phases, or at least this is not appreciable with the characterization techniques used. It is also noticed that the calculated mean crystallite size for both phases of the nanocomposites (NiO and GDC) exhibited merely equal values for the HM and BM methods, around 150 and 40 nm for NiO and GDC phase, respectively. However, the average-size values obtained for the CM method were smaller at 50 and 20 nm for NiO and GDC, respectively. This higher crystallinity matched well with the higher diffraction peak intensity observed in Figure 1 and demonstrated that the ball milling at low revolutions per minute did not have enough energy to decrease the crystallite size but helped to homogenize the two phases and to separate aggregates. Such growth of crystallite size for both phases in the NiO-GDC nanocomposite obtained by the CM method was not observed, being much lower than the pure phases. Therefore, it seems that during the phase formation by combustion, there are processes that inhibited the phase growth without the control of each one of them, especially in the NiO phase. This is expected with the immediate effect of having ceramic powders with a better dispersion, and therefore, we would expect a more homogeneous microstructure, with Ni particles hindered and a homogeneous distribution of the metal particles within the ceramic matrix [34,38], which is one of the key factors in this work.

**Figure 2:**
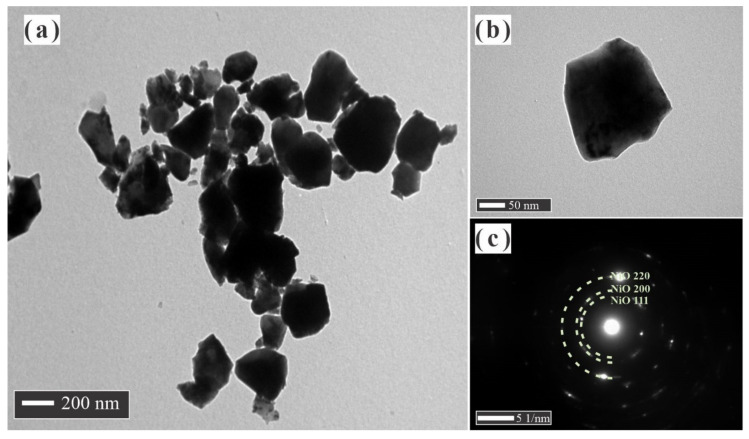
(**a**,**b**) TEM images and (**c**) SAED pattern of NiO powders.

**Figure 3:**
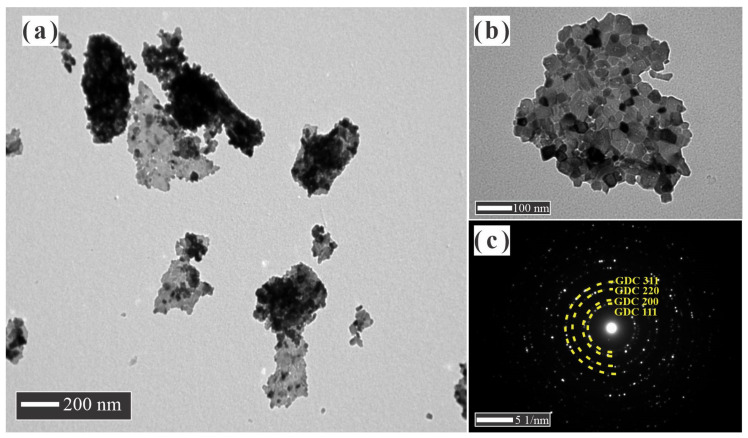
(**a**,**b**) TEM images and (**c**) SAED pattern of GDC powders.

**Figure 4:**
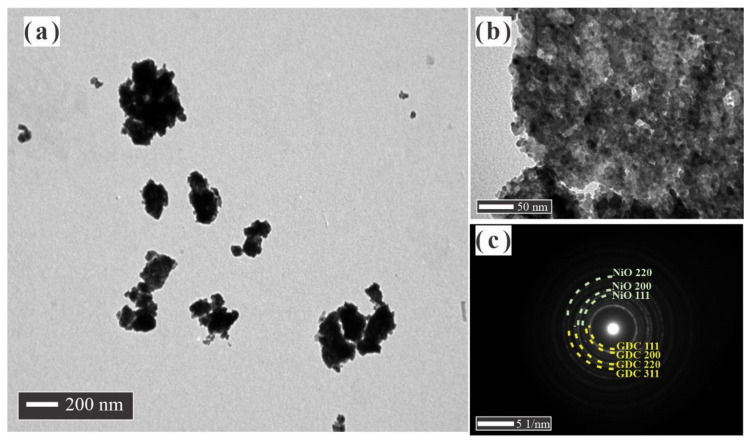
(**a**,**b**) TEM images and (**c**) SAED pattern of NiO-GDC nanocomposites obtained by CM method.

**Figure 5:**
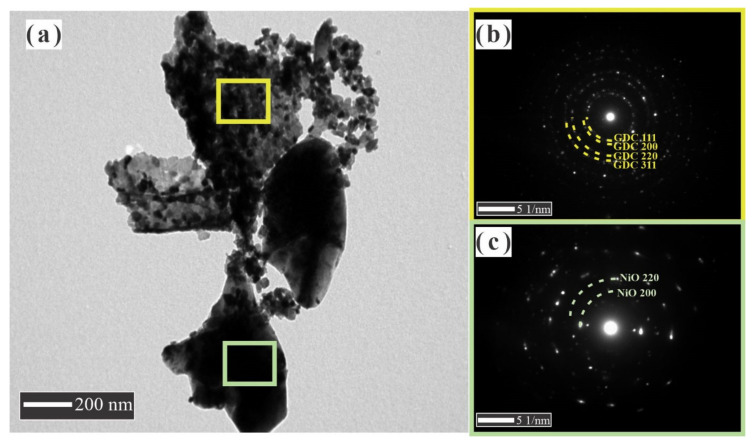
(**a**) TEM image and (**b**,**c**) spot SAED patterns of NiO-GDC nanocomposites obtained by conventional BM method.

The morphological features of NiO, GDC, and NiO-GDC nanocomposite powders obtained by cation and conventional mixing were examined by transmission electron microscopy (TEM) and the obtained morphologies are shown in Figure 2, Figure 3, Figure 4 and Figure 5. It is appreciated that a huge difference between the powders was observed. NiO powder is composed of thickened and sintered particles around 0.5 μm in average size. The porosity in these particles is not evident; rather, they are very smooth and well-formed crystals with high growth (Figure 2a,b). It is in an accordance with the values obtained by the XRD patterns and the surface area by adsorption isotherms where the specific area value is of the order of <1 m^2^·g^−1^. The SAED pattern obtained is the typical pattern for crystalline particles with large crystals (Figure 2c). The GDC powder morphology is completely different from the obtained NiO powder (Figure 3a,b). These particles are thin and similar to flakes, with an average size from few nm to around 0.5 μm; at the same time, large particles were formed by the coalescence of smaller crystallites, around 10 and 30 nm in size. Further, a large amount of porosity was observed in each particle, which is much more evident in the TEM image (Figure 3b). It is in an accordance with the values obtained by the XRD and the surface area by adsorption isotherms where the specific area value is of the order of 22 m^2^·g^−1^. In this case, well-formed crystals were observed with a smaller growth than the NiO crystals. In addition, because these particles are very thin, their size could be reduced by the ball mill mixing process. The SAED pattern showed a large amount of spotting, due to the number of crystallites that could be near the site of the incident beam because of its smaller size (Figure 3c).

The morphology of the NiO-GDC nanocomposite powder obtained by the CM method is similar to the observation in the GDC powder, with large particles of NiO and small GDC particles (Figure 4a,b). We assume that the NiO phase is well dispersed in the GDC phase with a particle size around 30 nm for both phases. Typically, the grains are composed of several crystallites, however, and in this case, one grain is composed of one crystallite, which is in accordance with the values obtained by XRD. Particles with many pores were observed, and the surface area values obtained by adsorption isotherms showed a specific area value of 9 m^2^·g^−1^.

Well-formed crystals with controlled growth in both phases and high dispersion and homogeneity were observed. It is also necessary to point out that there are few areas where small domains or clusters of NiO appear, which can reach the order of approximately 100 nm due to NiO’s high interfacial energy; therefore, they tend to grow and agglomerate. The SAED pattern showed a large number of diffraction rings very closed between them, which indicates that there are two phases (Figure 4c). On the other hand, the ring’s intensity is lower than the NiO and GDC SAED pattern, which confirmed that we are facing a small number of crystallites that diffracted in more directions and with less intensity due to their smaller size, which supports what was mentioned above about well-dispersed phases. The rings in the SAED pattern were easily associated with the corresponding crystallographic planes of GDC (yellow) and NiO (red), GDC (111), GDC (200), NiO (111), NiO (200), GDC (220) and GDC (311), and NiO (220), whose interplanar distances correspond to 3.173, 2.773, 2.465, 2.119, 1.944, 1.653, and 1.499 Å, respectively. The values were consistent with those corresponding to their pure phases indexed in the (GDC JCPDS No.: 75-0161) and (NiO JCPDS No.: 78-0643). As expected, the NiO-GDC nanocomposites obtained by the BM method, unlike those obtained by the CM method, showed a low dispersion of the NiO particles in the GDC phase (Figure 5a). The ball milling process did not reduce the size of the thick NiO particles, and it seems that some GDC particles, which were thinner, were broken. Each phase in the NiO-GDC nanocomposite keeps the properties of its original phase, which is easy to observe in the TEM micrograph. The surface area obtained by adsorption isotherms is 10 m^2^·g^−1^, clearly showing that this porosity is provided exclusively by the GDC phase. The SAED pattern shown in Figure 5a,b shows two zones of a particle aggregate, where thicker particles correspond to NiO and the thinner particles formed by the smaller crystallites correspond to GDC.

### 3.2. Characterization of the Sintered Discs

**Figure 6:**
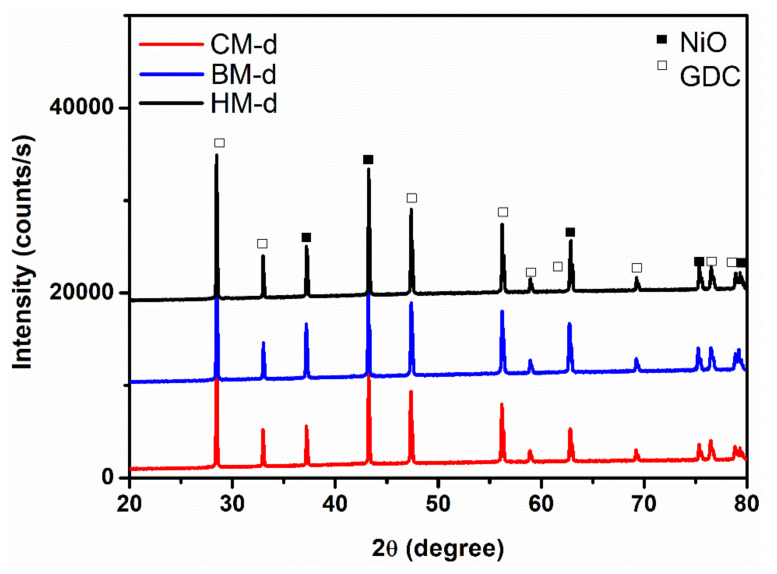
XRD patterns of the NiO-GDC sintered discs obtained by CM, BM, and HM methods (d represents the sintered disc).

The X-ray diffraction (XRD) patterns of the sintered discs obtained after compaction and sintering of the NiO-GDC calcined powders obtained under the different processing methods (BM, CM, and HM) are shown in Figure 6. Similar to the powder analysis, the phases present in these diffraction patterns were identified by the search-match technique using the JCPDS database, and the microstructural parameters were studied by the Rietveld refinement analysis, the results of which are presented in Table 3. As determined in Section 3.1, the absence of any additional peaks confirmed the successful formation of the well crystalline NiO-GDC composites, free of any secondary phases, which guarantees the stability of the phases. Remarkably, narrowed peaks were observed for all samples (CM-d, BM-d, and HM-d, shown in Figure 6) by comparing with their precursors powders (CM-p, BM-p, and HM-p, Figure 1), which is due to the higher crystallinity of the phases by the high temperature at which the compacted discs were subjected to sintering. This higher crystallinity is also confirmed by the crystallite size determination by the Rietveld analysis.

**Figure 7:**
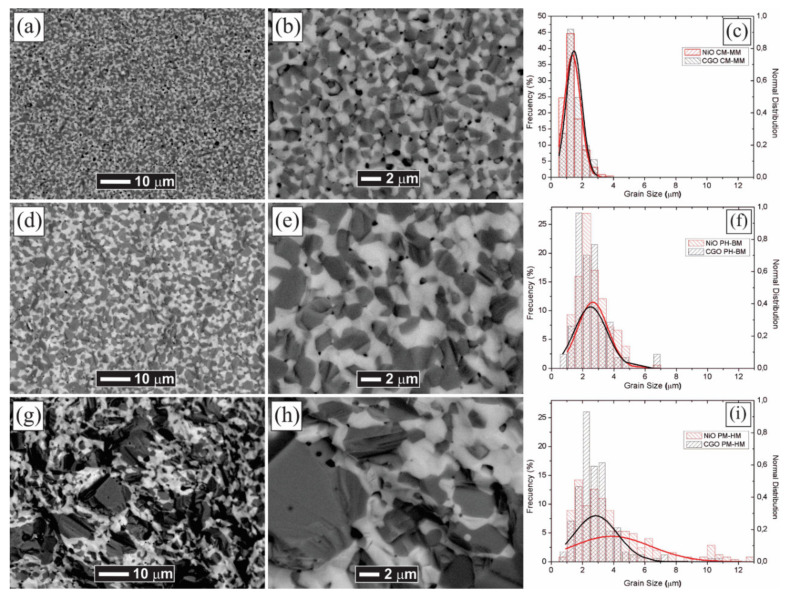
SEM images of the fracture surfaces for the sintered samples processed through (**a**,**b**) CM, (**d**,**e**) BM, and (**g**,**h**) HM methods. Grain size distribution for the sintered samples processed through (**c**) CM, (**f**) BM, and (**i**) HM methods.

The SEM images of the sintered discs microstructure obtained by the different methods used are shown in Figure 7. The dark grey and white phases correspond to NiO and GDC, respectively, and are easily distinguished using the BSE detector. As estimated, despite the high temperature and long-time conditions used in the sintering process, the three starting methods yield different microstructures with highly dispersed and well-connected NiO and GDC grains for the CM method. The grain size distributions of the NiO-GDC composites were obtained by employing image analysis using the Image-J software. With the CM method, the mean grain sizes for NiO and GDC, which were determined as 1.4 and 1.5 μm, respectively, with a standard deviation of 0.5 μm in both cases, which is observed in Figure 7c. This makes CM by far the best mixing condition for creating a large TPB, NiO-NiO electronic conduction, and GDC-GDC oxygen conduction. On the other hand, between the conventional mixing methods, the BM method generated a better phase homogenization than the HM method, with a mean grain size for NiO and GDC of 2.6 and 2.5 μm, respectively, and a standard deviation of 1.0 μm in both cases, as shown in Figure 7f. Furthermore, the HM method led to the worst condition, with a highly heterogeneous microstructure with an average grain size for NiO and GDC of 3.9 and 2.9 μm, and a standard deviation of 2.5 and 1.4 μm, respectively. This possibly caused some disconnected regions, shown by clusters of a large grain of both phases up to 20 μm (Figure 7i).

The deformation caused by the application of the load on the solid structures is considered as an amount of hardness in the materials. The characteristics of the NiO-GDC discs obtained under the different processing methods are presented in Table 4. As-synthesized powders of pure NiO and GDC were compacted and sintered under the same conditions for further comparison, and their measured hardness values correspond to 3.21 ± 0.26 and 7.58 ± 0.49 GPa, respectively. The point and interval were estimated with the confidence of 95% of medium hardness value. The greater hardness value of the NiO-GDC composite obtained by the CM method is associated with the smaller grain size of the two phases and its higher homogeneity (Figure 7a–c).

The theoretical densities of the samples were calculated according to the rule of mixtures, using the density values of 6.67 and 7.16 g·cm^−3^ for NiO and GDC, respectively; then, the volume content of each phase was calculated using the wt. % obtained by the Rietveld refinements presented in Table 3. The relative densities of all the composites are similar, and their values are in an accordance with a previously reported work by Grilo et al. [34]. It is well known that the sintered density is dependent on the morphology and size of the powders, as well as sintering parameters, and that powder mixtures with smaller particle sizes are easily densified rather than large particles under the same sintering conditions [48]. On the other hand, the nanocomposites processed by the CM method showed larger grain sizes, which are associated with higher porosity, but it also presented a percentage of well-dispersed fine grains. This microstructure is associated with the large difference between the starting NiO and GDC nanocomposite powders. In general, for all methods, the GDC presented very similar grain sizes, and its high sintering temperature (~1500 °C) prevents the full densification of the NiO-GDC materials [49].

**Figure 8:**
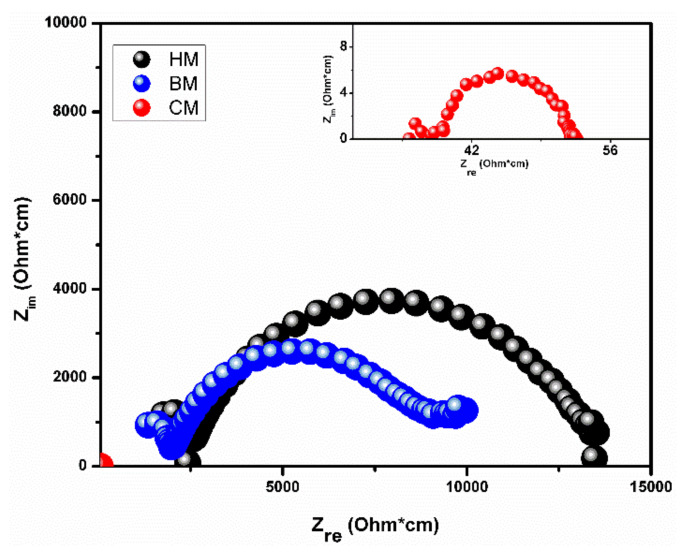
Impedance spectra at 350 °C for the sintered samples processed through CM (red), BM (blue), and HM (black) methods.

The impedance spectra obtained at 350 °C for the samples made by the three mixing methods are shown in Figure 8. The impedance spectra show two arcs at high and low frequencies. In a single phase, the high-frequency arc is associated with the grain or bulk resistance, while the low frequency is related to grain boundary resistance. However, the direct separation of contributions is not possible, since GDC and NiO also provide parallel ionic and electronic pathways, respectively. Grilo et al. [34] proposed a reasonable model that includes the contribution of a parallel pathway of each phase. For a proper study of impedance spectra, it is necessary to consider the dispersion of the conductive phase NiO. The notable difference in the size of the arcs between CM and conventional methods (BM and HM) might be attributed to the presence of a good percolating electronic pathway in the CM samples, in contrast with high impedance obtained in both BM and HM. The microstructure of the CM samples (Figure 7a,b) shows a refined and similar grain size between NiO and GDC phases. This distribution would increase the probability of NiO conduction pathway, which has the main influence on conductivity in the low range of temperature (350 °C). In contrast, hand mixing shows an excessive growth in the NiO phase (Figure 7g,h), which caused a reduction in the available surface area of the metal catalyst, thus reducing the number of active catalytic sites and ultimately increasing the activation polarization. When grain size is too large, it causes a disconnection between the metal catalyst particles, which decreases the electrical conductivity, generating an increase in the ohmic polarization [50,51,52,53].

**Figure 9:**
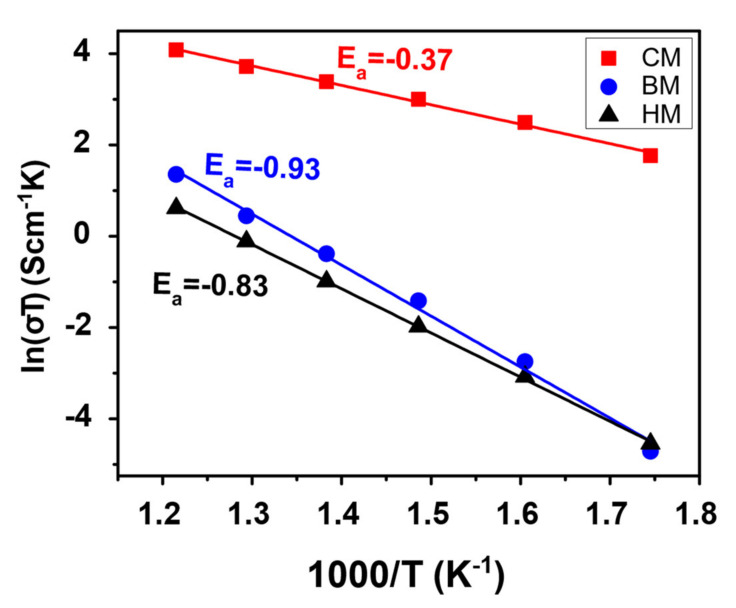
Arrhenius plots of total conductivity for the sintered samples processed through CM (red), BM (blue), and HM (black) methods.

The total electrical conductivity was obtained from the low-frequency intercept of the impedance spectra with the real axis. The total conductivity (σ) was calculated by using the Arrhenius type dependence: σT=σ0e−f0EaRT, where T is the temperature, $\sigma_{0}$ is a pre-exponential term and R is the gas constant. The activation energy ($E_{a}$) was calculated from the slope of the Arrhenius curve (Figure 9), with values of 0.37, 0.83, and 0.93 for CM, HM, and BM methods, respectively. The activation energy helped to elucidate the dominant conducting mechanism in the composite. The activation energy value for the CM method is close to the NiO phase, while the activation energy for HM and BM methods is near to GDC. Considering these values of activation energy and the difference in conductivity ranges, the samples under the CM method would have a conduction mechanism dominated by the percolation of the NiO phase, while the samples under mechanical mixing, the conductivity is dominated by the GDC phase. Therefore, the NiO-GDC nanocomposite powder obtained by solution combustion using a CM method not only allows for improving the microstructure, but also for mechanical and electrical behaviors. Meanwhile, the obvious difference between the two conventional BM and HM microstructures observed in Figure 7d,g, respectively, seems not to greatly affect the dominating conduction mechanism, maintaining a similar behavior.

## 4. Conclusions

The results show the advantages in terms of time, scalability, reduced crystallite size, and better dispersion between the NiO and GDC phases for NiO-GDC (65:35 wt. %) nanocomposites synthesized through the fast solution combustion by the chemical method (CM) compared to the traditional methods such as the ball-milling (BM) and hand-mortar and pestle (HM) method. Furthermore, the X-ray diffraction results showed that nanocomposite obtained by the CM method was free to any secondary phase, similar to the traditional methods; therefore, there were no drawbacks to using the CM method. In addition, the synthesized nanocomposite led to high-phase percolation, which improved the electrical properties with activation energy (0.37 eV), close to the electronic NiO conducting mechanism and compared with both traditional methods, where the conducting mechanisms were close to the ionic GDC. Based on these results, using NiO-GDC nanocomposite obtained by CM method as anode precursor material, is possible to fabricate anodes with better stability after redox cycles, electrical and mechanical properties for SOFCs.

## Figures and Tables

**Table 1:** XRF results of NiO, GDC, and nanocomposite powders obtained by CM and BM method.

Samples	Phases	Elements	mol. %		wt. %	
			Stoichiometric	Calculated	Stoichiometric	Calculated
NiO	NiO				100	99.9
GDC	GDC	Ce	0.9	0.906	100	99.7
		Gd	0.1	0.094		
NiO-GDC (CM-p)	NiO				65	64.6
	GDC	Ce	0.9	0.905	35	35.3
		Gd	0.1	0.095		
NiO-GDC (BM-p)	NiO				65	65.6
	GDC	Ce	0.9	0.910	35	34.4
		Gd	0.1	0.090		

**Table 2:** Quantitative phase analysis of the nanocomposite performed by Rietveld refinement. The quality refinement parameters; GOF: goodness of fit; D: mean crystallite size and ˂ε^2^˃^1/2^: root mean square (r.m.s.) of the microstrain are also presented.

Samples	Rietveld Refinement Results					
	R_wp_	GOF	Phase	wt. %	D (nm)	˂ε^2^˃^1/2^
**HM-p**	2.0	1.6	NiO	63.2	141	6.4 × 10^−4^
			GDC	36.8	38	1.0 × 10^−3^
**BM-p**	2.0	1.6	NiO	64.4	141	7.6 × 10^−4^
			GDC	35.6	36	1.0 × 10^−3^
**CM-p**	2.0	1.6	NiO	63.6	47	5.0 × 10^−4^
			GDC	36.4	19	1.7 × 10^−3^

**Table 3:** Quantitative analysis of phases of the sintered discs analyzed from the Rietveld and the quality refinement parameters. GOF: goodness of fit; D: mean crystallite size; ˂ε^2^˃^1/2^: root mean square (rms) of the microstrain.

Samples	Rietveld Refinement Results					
	R_wp_	GOF	Phase	wt. %	D (nm)	˂ε^2^˃^1/2^
**HM**	8.7	1.9	NiO	63.9	1750	3.8 × 10^−4^
			GDC	36.1	2137	4.4 × 10^−4^
**BM**	5.3	1.9	NiO	65.3	1377	4.4 × 10^−4^
			GDC	34.7	1414	5.9 × 10^−4^
**CM**	7.5	1.9	NiO	59.7	1242	3.5 × 10^−4^
			GDC	40.3	1697	5.2 × 10^−4^

**Table 4:** Characteristics of the NiO-GDC discs obtained under the different processing methods (BM, CM, and HM).

Features	CM	BM	HM
Hardness (GPa)	6.04 ± 0.17	5.96 ± 0.34	5.41 ± 0.21
Theoretical density (g·cm^−3^)	6.86	6.83	6.84
Measured density (g·cm^−3^)	6.09 ± 0.01	6.10 ± 0.03	6.18 ± 0.04
Relative density (%)	88.9 ± 0.3	89.1 ± 0.1	90.4 ± 0.5

## Data Availability

The data presented in this study are available on request from the corresponding author. At the time the project was carried out, there was no obligation to make the data publicly available.
